# Supplementary material for: Complexome profiling on the Chlamydomonas lpa2 mutant reveals insights into PSII biogenesis and new PSII associated proteins
Source: J Exp Bot. 2021 Aug 26;73(1):245–62. doi: 10.1093/jxb/erab390 (PMC8730698; doi:10.1093/jxb/erab390)
Supplement: erab390_suppl_Supplementary_Dataset_S1 [file erab390_suppl_supplementary_dataset_s1.zip › Supplemental Dataset 1 - Excel List and all profiles/plots/RPS19_Cre02.g106600.html]

Trivial name: RPS19  
  
Euclidean distance: 2812.10  
Mean Intensity (WT): 58.62  
Mean Intensity (Mut): 123.44  
Distance: 22.78  
  
MapMan: protein.synthesis.ribosomal protein.eukaryotic.40S subunit.S19  
  
p value of intensity sums Welch test: 0.2916
